# Adolescent Interpersonal Behaviours and Mental Health Across Two Swedish Cohorts: 15-Year Trends and One-Year Bidirectional Associations in a Mixed-Methods Study

**DOI:** 10.1186/s13034-025-00913-5

**Published:** 2025-05-21

**Authors:** Moa Nilsson, Benjamin Claréus, Jonas Bjärehed, Marlene Bjärehed, Daiva Daukantaitė

**Affiliations:** 1 Department of Psychology, Lund University, Lund, 221 00 Sweden; 2 Department of Psychology, Kristianstad University, Kristianstad, 291 88 Sweden; 3 Department of Primary Teacher Education, Kristianstad University, Kristianstad, 291 88 Sweden

**Keywords:** Adolescence, Online, Bullying, Aggression, Victimization, Time-trends, Longitudinal, Mixed-methods, Content analysis, Mental health

## Abstract

**Background and Aims:**

Adolescent mental health has declined in recent decades. Few studies have explored whether shifts in interpersonal behaviours– both in-person and online– can help explain this decline. Using data from two Swedish cohorts (2007–2008, 2023–2024), we examined (1) 15-year time trends (2007–2008 vs. 2023–2024) in interpersonal behaviours (victimization, aggression, prosocial behaviours) and mental health (externalizing/internalizing problems); (2) one-year bidirectional associations between them; (3) the unique role of cybervictimization/cyberaggression in relation to mental health; and (4) adolescents’ accounts of online experiences that made them ‘feel bad’.

**Methods:**

Two longitudinal school cohorts of Swedish adolescents (2007–2008: *N* = 911–987, *M*_age_ = 13.71–14.76 years; 2023–2024: *N* = 768–806, *M*_age_ = 13.89–14.89 years) completed self-report measures of interpersonal behaviours and mental health. In 2023–2024, a subset of adolescents (*n* = 127, 16.5%) answered open-ended questions about negative online experiences.

**Results:**

From 2007 to 2008 to 2023–2024, prosocial behaviours moderately declined for girls and boys, while internalizing problems increased moderately for girls and weakly for boys. Meanwhile, victimization, aggression, and externalizing problems increased at a weak-to-moderate level among girls. Victimization and being treated well by others showed moderate bidirectional relationships with mental health. Cybervictimization/cyberaggression had weaker associations with mental health than did in-person behaviours. According to the qualitative analysis of negative online experiences, adolescents reported harassment, social exclusion, perceived standards/expectations, time-consuming activities, and exposure to distressing content.

**Conclusions:**

Deteriorating adolescent mental health over the past 15 years has occurred alongside rising interpersonal difficulties. The findings further suggest a reciprocal relationship between social challenges and mental health, with hostile online environments amplifying– but not primarily driving– these issues. A holistic perspective that accounts for both in-person and online experiences is essential to better understand and support adolescent well-being.

**Supplementary Information:**

The online version contains supplementary material available at 10.1186/s13034-025-00913-5.

Adolescent mental health has deteriorated significantly since the early 2000s, according to research conducted in the global North [1–11]. This decline has been particularly pronounced among girls [1, 6, 12, 13]. According to Bronfenbrenner’s framework [14], the decline is understood as driven by a complex interplay of factors operating from micro- to macrosystemic levels. One micro-level factor widely recognized as influencing adolescent mental health (particularly in the 12–17 age range) is the nature and quality of adolescents’ relationships and interpersonal interactions within their immediate context (e.g., peers, friends, and family). Within these relationships, aggression and victimization stand out as clear indicators of poor relational quality and negative interpersonal interactions [15]. Aggression refers to behaviours– verbal, physical, or relational– intended to harm or dominate another individual [16]; victimization occurs when a person is subjected to these harmful actions. Both can have far-reaching consequences for adolescents, including an increased risk of concurrent and long-term mental health problems [7, 17–23]. Additionally, such experiences may hamper the development of crucial social skills and undermine the formation of meaningful relationships later in life [15].

In the present study, we utilize longitudinal data from two Swedish school cohorts (2007–2008 and 2023–2024) to investigate how offline and online interpersonal behaviours relate to adolescent mental health over time. First, we examine 15-year trends in aggression, victimization, prosocial behaviours, and mental health problems. Second, we explore one-year bidirectional associations between interpersonal interactions and externalizing/internalizing problems. Third, we assess the unique contributions of cybervictimization and cyberaggression to mental health outcomes in the 2023–2024 cohort, in comparison to in-person experiences. Finally, we analyse adolescents’ written descriptions of negative online experiences using qualitative content analysis, to better understand the emotional impact of digital interactions and to contextualize the quantitative findings. By examining these distinct yet interrelated forms of negative interpersonal interactions, we seek to deepen understanding of how relational dynamics influence adolescent mental health and well-being over time.

## Time trends in interpersonal behaviours and mental health

Over the past two decades, mental health problems such as anxiety and depression have risen markedly among European adolescents, according to the WHO-coordinated Health Behaviour in School-aged Children (HBSC) study [2, 4, 6, 9, 11]. In the Nordic region, estimates by Eriksson and Stattin [4] suggest that the prevalence of such problems in Sweden has at least doubled between 2002 and 2022. Gender-specific analyses indicate that this increase has been especially pronounced among girls [4, 6].

While numerous studies have established longitudinal links between adolescent mental health and interpersonal behaviours– including victimization, aggression, and prosocial behaviour [2, 4, 6, 8, 9, 11]– few, if any, have examined whether these phenomena have changed in parallel over time. Understanding the parallel trends in mental health and peer interactions is crucial, particularly as digital platforms have become a central arena for adolescent social life [10, 11, 18].

In Sweden, schools are legally mandated to prevent and address degrading treatment and harassment, including bullying [24]. However, they are not required to implement specific anti-bullying programs. Nevertheless, some schools and municipalities have voluntarily adopted evidence-based interventions such as the Olweus Bullying Prevention Program [25] and KiVa [26], which are widely used across Nordic countries and have been evaluated internationally [24, 25, 27]. Despite these initiatives, recent studies suggest that bullying and aggression have increased in Sweden over the past decade [10, 11, 18], with similar trends being observed in neighbouring countries like Norway (e.g [28]).

## The bidirectional relationships between interpersonal interactions and adolescent mental health

Adolescent mental health is influenced by a variety of interpersonal interactions, including aggression, victimization, and prosocial behaviour. Aggressive behaviours manifest both directly and indirectly, ranging from physical violence to social exclusion and name-calling [16], and take place in online as well as in in-person settings. Both aggression and victimization also encompass bullying, which is defined by intention, repetition, and a power imbalance between victim(s) and aggressor(s) [29]. Notably, being victimized in adolescence has been associated with an increased risk of internalizing problems (e.g., emotional problems, depression, anxiety) up to 17 years later, as well as reduced life satisfaction [7, 17, 19–23]. A review by Christina et al. [30] suggests that the association is likely bidirectional– in other words, internalizing problems and victimization have a reciprocal relationship. This reciprocity applies to both between-person and within-person developments, discussed further by Laninga-Wijnen et al. [31] Specifically, between-processes suggest that individuals who score higher on victimization relative to others are also likely to score higher on internalizing problems compared to their peers. In contrast, within-person processes refer to how an individual’s own internalizing problems are correlated with their own experiences of victimization. Besides internalizing problems, victimization can lead to externalizing problems such as defiant behaviour, dishonesty, and irritability [32, 33].

By contrast, prosocial behaviours involve acting kindly toward others (e.g., offering help or including peers in activities) and can foster social support and connectedness among adolescents [12, 34]. The studies reviewed by Schacter et al. [35] found that prosocial behaviour (in terms of friendship quality) might weaken as well as strengthen the negative effect of victimization on mental health. Accordingly, the authors emphasize the need for further research on how prosocial behaviours have a protective effect on the relationship between victimization/aggression and mental health. In the present study, we focus on the reciprocity of prosocial behaviour over time rather than its moderating role. For example, prior research indicates that adolescents experiencing mental health problems are less likely to engage in prosocial behaviour themselves [36], highlighting the value of studying these behaviours as outcomes within a bidirectional framework.

### Cybervictimization, cyberaggression, and adolescent mental health

Adolescents today navigate online environments that expose them to new forms of social interaction– including various forms of cybervictimization and cyberaggression– that differ markedly from those experienced by youth in the early 2000s. Qualitative studies on adolescents’ online experiences [37–39] reveal the multifaceted nature of these behaviours, which can include social pressure, exclusion, name-calling, and abusive messaging, among other behaviours [40].

As with in-person victimization, cybervictimization has been associated with heightened internalizing problems, including depression [41]. However, adolescents frequently report that cybervictimization co-occurs with negative treatment by others in person [37, 38, 42]. This observation is supported by quantitative research indicating that up to two-thirds of adolescents who experience in-person victimization are also victimized online [41]. As argued by Olweus and Limber [43], understanding the effects of cyber- and in-person victimization requires studying both phenomena concurrently. One such study by Salmivalli et al. [41] found that cybervictimization alone did not contribute to increased symptoms of anxiety and depression, compared to co-occurring cyber- and in-person victimization. More recent studies have yielded similar findings [45, 46]. A recent meta-analysis further suggests that victimization exerts the most detrimental impact on adolescent mental health when it occurs both online and in-person [41].

## The present study

The present study addresses four overarching objectives to better understand how interpersonal behaviours, both offline and online, associate with adolescent mental health over time.

First, we explore time trends in mean scores of aggression, victimization, prosocial behaviours, and externalizing/internalizing problems among Swedish adolescents from 2007 to 2008 to 2023–2024. Based on studies ending before the 2020s [7, 20, 22, 23] and focused on Swedish samples in particular [18], we hypothesize that adolescents in the 2023–2024 cohort, particularly girls [see 4], report higher levels of victimization/aggression and internalizing problems compared to those in 2007–2008. While we were unable to identify any cohort study where the last data collection was in 2020 or later that assessed time trends in externalizing problems and prosocial behaviours, we anticipate that any observed trends in these variables will align with those in victimization/aggression and internalizing problems, given their established interrelations [30–33].

Second, we investigated bidirectional associations between interpersonal interactions and mental health. We include both negative interpersonal experiences and prosocial behaviour (i.e., being treated well by others and treating others well), as prior work suggests they may buffer or moderate the impact of aggression and victimization on mental health problems [36, 40, 47–49]. In line with previous research, we expected to find bidirectional associations between victimization/aggression and prosocial behaviours with mental health problems [12, 30, 31, 34].

Third, we assess the unique contribution of cybervictimization and cyberaggression in adolescent mental health in the 2023–2024 cohort. Specifically, we investigate whether these online experiences explain any additional variance in externalizing and internalizing symptoms, beyond that explained by in-person victimization/aggression and prosocial behaviours. While we anticipate that cybervictimization and cyberaggression account for some variance in mental health problems, we expect their effects to be smaller than those of in-person victimization/aggression [44–46].

Finally, we incorporate a qualitative component to capture adolescents’ own perceptions of emotionally negative online experiences. Using a content analysis of open-ended responses from a subset of 2024 participants, we explore the types of online interactions adolescents report as making them ‘feel bad’. This qualitative approach helps to contextualize the quantitative findings and capture nuanced experiences that quantitative methods alone may overlook [50, 51]. Specifically, the inductive nature of this analysis enabled us to identify potential limitations in how cybervictimization is operationalized. It also provides insight into which online experiences adolescents themselves view as most impactful, providing a lived-experience perspective on the digital antecedents of negative emotional reactions.

## Method

### Participants

The current study is part of a larger longitudinal research project with data collections in 2007 and 2008 (period 1: *N* = 911–987, response rate = 90.0–93.0%, *M*_age_ = 13.7–14.8 years, 50.3–51.1% girls), and 2023 and 2024 (period 2: *N* = 768–806, response rate = 75.2–79.2%, *M*_age_ = 13.9–14.9, 48.1–48.8% girls). A detailed overview of the samples is provided in Table 1. Eligible participants at baseline (2007, 2023) included students in Grade 7 and Grade 8 in all public and private schools within a southern Swedish municipality, and one year later at follow-up (2008, 2024), included all Grade 8 and Grade 9 students in the same municipality.

**Table 1:** Sample demographics and measurement internal consistency at different data collections

		Period 1: 2007 − 2008			Period 2: 2023 − 2024	
		2007	2008		2023	2024
*n* participating schools		5	6		7	8
*N* participants		991	987		806	768
Response rate– %		93.0%	90.0%		79.2%	75.2%
Age– *M* (*SD*)		13.71 (0.68)	14.76 (0.69)		13.89 (0.75)	14.89 (0.73)
Grade– *n* (%)						
	Grade 7	507 (51.16%)	-		409 (50.75%)	-
	Grade 8	484 (48.84%)	513 (51.97%)		397 (49.25%)	379 (49.35%)
	Grade 9	-	474 (48.03%)		-	389 (50.65%)
Gender– *n* (%)						
	Girls	496 (50.30%)	501 (51.10%)		388 (48.14%)	374 (48.77%)
	Boys	491 (49.70%)	480 (48.90%)		404 (50.12%)	381 (49.67%)
	Other/Do not want to disclose^a^	-	-		14 (1.74%)	12 (1.56%)
Background– *n* (%)						
	Sweden	836 (84.62%)	833 (84.96%)		584 (72.46%)	580 (75.52%)
	Foreign^b^	152 (15.38%)	147 (15.04%)		222 (27.54%)	188 (24.48%)
PANIBI-SF– Cronbach’s *α*						
	Treating others well	0.66	0.64		0.63	0.68
	Aggression	0.78	0.84		0.84	0.87
	Cyberaggression	-	-		0.79	0.86
	Being treated well by others	0.66	0.64		0.63	0.66
	Victimization	0.84	0.85		0.87	0.86
	Cybervictimization	-	-		0.61	0.82
SDQ-s– Cronbach’s *α*						
	Externalizing problems	0.58	0.61		0.62	0.56
	Internalizing problems	0.68	0.69		0.77	0.77

The additional schools started operations for students in Grades 7 − 9 between data collections. Discrepancies in gender between the first and second data collection was resolved by aligning the first observation with the last. Externalizing problems were operationalized as conduct problems, and internalizing problems as emotional symptoms. PANIBI-SF = Positive and Negative Interpersonal Behaviours Inventory-Short Form; SDQ-s = Strength and Difficulties Questionnaire, self-report version

^a^ Not an available response option in 2007 − 2008. ^b^ Defined as the participant either (i) being born abroad with at least one parent born abroad as well, (ii) or being born in Sweden with both parents being born abroad

The municipality wherein the data was collected had about 47 000 inhabitants in 2023, and its population increase since 2007 (14.8%) was comparable to the increase in the overall Swedish population (15.1%). Additionally, official data from Statistics Sweden suggest that, relative to Sweden as a whole, the municipality was slightly more rural (80.2% vs. 84.4% living in urban areas in 2007, and 85.5% vs. 87.6% in 2023), had a lower mean income level per year (207 100 SEK vs. 224 400 SEK in 2007, and 327 000 SEK vs. 348 600 SEK in 2023), and had a lower level of education (15.7% vs. 22.2% had a university education in 2007, and 22.3% vs. 28.9% in 2023).

## Measures

### Positive and negative interpersonal behaviours inventory– short form (PANIBI-SF)

The PANIBI, developed by Lundh et al. [21], originally consisted of 40 items designed to measure aggression, victimization, and prosocial behaviour. Its design incorporates two key principles to ensure balanced and comprehensive assessment. First, participants were asked about aggressive behaviours they experience from others (i.e., being a victim) before being asked about their own aggressive behaviours towards others (i.e., being aggressive). This sequencing was designed to reduce the likelihood of defensiveness when participants answered questions about their own aggression. Second, the items measuring aggressive behaviours are complemented by items measuring prosocial behaviours, reducing the emphasis on aggression. To reflect these principles, the PANIBI was symmetrically structured along two dimensions: valence (positive vs. negative) and direction of behaviour (self-to-others vs. others-to-self).

This 40-item version of the PANIBI was administered in 2007–2008. Prior to data collection in 2023–2024, the PANIBI was revised and shortened. Therefore, the current study only includes analyses based on the 16 core items that were identical across both periods. These core items were selected based on their endorsement rates, factor loadings, face validity, and construct coverage. In 2023–2024, we also included 8 items from the European Bullying Intervention Project Questionnaire [52] were included to assess cyberaggression and cybervictimization.

The final version was labelled PANIBI-SF. To summarize, the PANIBI-SF measures the following constructs using a scale ranging from 1 (*never*) to 5 (*very often*): (1) (cyber)victimization, which refers to being the target of aggressive behaviour, either in-person or online (e.g., ‘How often does it happen that someone hits or kicks you?’ or ‘How often does it happen that someone posts embarrassing videos or pictures of you on the internet?’); (2) being treated well by others, which captures experiences of positive, prosocial actions from others (e.g., ‘How often does it happen that someone gives you a hug?’); (3) (cyber)aggression, which refers to perpetrating aggressive behaviour, either in-person or online (e.g., ‘How often does it happen that you hit or kick someone?’ or ‘How often does it happen that you post embarrassing videos or pictures of someone on the internet?’); and (4) treating others well, which reflects engaging in positive, prosocial actions towards others (e.g., ‘How often does it happen that you give someone a hug?’) (see Supplementary Appendix Tables A1 and A2).

As shown in Table 1, the internal consistency of the PANIBI-SF ranged from *α* = 0.63-0.68 for prosocial behaviours, *α* = 0.78-0.87 for victimization/aggression, and *α* = 0.76–86 for cybervictimization/cyberaggression. The lower Cronbach’s *α* values for the subscales measuring prosocial behaviour was considered acceptable because they include fewer items. Additionally, analyses in Table A3 in the Supplementary Appendix supported a good factor structure (CFI ≥ 0.952; RMSEA ≤ 0.073; SRMR ≤ 0.036; c.f [53]), and at least metric invariance between 2007 and 2008 and 2023–2024 (|Δ|CFI ≤ 0.017;|Δ|RMSEA ≤ 0.009;|Δ|SRMR ≤ 0.019; c.f [54]) for the PANIBI-SF.

## Strength and difficulties questionnaire– self-report version (SDQ-s)

The SDQ-s was developed by Goodman [55]. The subscale *conduct problems* (5 items) was used to operationalize externalizing problems, while *emotional problems* (5 items) was used to operationalize internalizing problems [c.f., 13]. Participants rank each item with a 3-point Likert scale (0 = *not true*, 1 = *somewhat true*, 2 = *certainly true*), which is summed for each subscale. Cronbach’s *α* values were *α* = 0.58-0.69 in 2007–2008, and *α* = 0.56-0.77 in 2023–2024 (cf., Table 1). The relatively low *α* values (*α* ≤ 0.62) for the conduct problems subscale are a recurrent issue in the literature, but it has not been shown to negatively impact scale validity [56]. Furthermore, as shown in Table A4 in the Supplementary appendix, at least metric invariance was supported between 2007 and 2008 and 2023–2024 (|Δ|CFI ≤ 0.016;|Δ|RMSEA ≤ 0.003;|Δ|SRMR ≤ 0.004; c.f [54]) for externalizing/internalizing problems.

## Negative online experiences

To further investigate negative online experiences among adolescents, respondents in 2024 were asked: ‘Have you ever felt bad because of social media?’ and ‘Have you ever felt bad because of gaming?’ (response options: *yes*, *sometimes*, or *no*). Participants who answered *yes* or *sometimes* were then asked to elaborate upon their experiences in text.

### Procedure

In Sweden, attending preschool and Grades 1–9 in either a public or private school is mandatory, where Grades 7–9 roughly correspond to ages 13–16 (the target age range of this study). Data collection in 2007–2008 and 2023–2024 was initiated by contacting the principals of the public and private schools within the municipality, who agreed to their school’s participation. Information about the study was then sent out to the parents/legal guardians, who had to contact the teachers or researchers if they did not consent to their child’s participation. The appropriate legal body had reviewed and approved of this passive parental consent procedure beforehand, and it was considered appropriate given the minor risks associated with participation [57] as well as necessary for ensuring sample representativeness [58]. Students themselves had to actively consent to participation, and could withdraw by telling the teachers/researchers, terminating the survey, or handing in a blank survey.

The questionnaire was administered by research assistants from Lund University in a classroom setting during regular school hours, with a teacher present. The research assistants verbally provided information about the project, the students’ right to withdraw, and confidentiality. The questionnaire contained additional measurements not included for analysis in the present study. In 2007–2008, the questionnaire consisted of 11 pages and was administered on paper. In 2023–2024, the questionnaire consisted of 10 pages and was administered digitally. Students’ responses were pseudonymized such that their answers at baseline and follow-up could be connected using a unique identifying code. During questionnaire administration, research assistants remained attentive to signs of distress among the students and were prepared to direct the students to the appropriate support resources if necessary.

### Data analysis

#### Mixed linear modelling

Research question 1 (analysing mean differences in the PANIBI-SF and SDQ-s across 15 years) and research question 2 (assessing one-year associations between interpersonal interactions and mental health) were addressed using restricted-maximum likelihood mixed linear modelling (MLM) as provided by *lme4* (version 1.1–35.3; [59]) and *afex* (version 1.3-1; [60]) for R (version 4.4.0). Prior to the analysis, missingness in occasional items was imputed with expectation–maximization before calculating the scale scores. Attrition at assessment (e.g., only answering at baseline or follow-up) was accounted for in the mixed modelling by including *all* participants in the analysis regardless of response status at baseline/follow-up (participant ID was set as the random intercept). Robust estimates that balance for residual non-normality were included to ascertain the reliability of the fixed effects [61]. When considerable differences between the robust and non-robust estimate were found, both were considered in interpreting the effect size.

For the models examining 15-year mean differences, period of data collection (0 = *2007–2008*, 1 = *2023–2024*) and participant gender (0 = *girls*, 1 = *boys*; *other/undisclosed* was set as case-wise missing due to small subsample sizes), and the interaction between the two were added as fixed effects. In case of significant interaction effects, the degree of estimated marginal change among girls and boys was post-hoc tested with Bonferroni corrections (using *emmeans*, version 1.10.6 [62]). Time of assessment (0 = *baseline*, i.e.,* 2007 or 2023*, 1 = *follow-up*, i.e.,* 2008 or 2024*) was included as a covariate to control for eventual regression towards the mean.

For the models testing one-year associations (i.e., bidirectionality and the relative contribution of cybervictimization/cyberaggression), all outcomes were entered as time-variant, such that the model predicted changes in the outcome as related to the fixed effects (entered as time-invariant). All one-year mixed linear models included participant gender and, when relevant, period of data collection as covariates to hold eventual outcome differences constant. The marginal *R*^2^ (as provided by *MuMIn*, version 1.47.5; [63]) was computed to examine the relative contribution of the fixed effects. Bidirectionality was evaluated using two separate models: one included interpersonal behaviours at baseline as predictors of change in mental health, and the other included mental health at baseline as the predictor of change in interpersonal behaviours. We tested for the relative importance of cybervictimization and cyberaggression in 2023–2024 (as online interactions were not assessed in 2007–2008) by hierarchical MLM and compared the models using the likelihood ratio tests.

Due to the increase in students reporting a foreign background from 2007 to 2008 to 2023–2024, we also ran all MLMs as described above with foreign background as a covariate (Supplementary Appendix Table A5–A7). As controlling for foreign background did not appreciably change any model estimates (|Δ|*R*^2^_marginal_ = 0.00-0.01) or beta coefficients (|Δ|*β* = 0.00-0.04), we report the unadjusted coefficients in the main body of the manuscript.

### Content analysis

To analyse the open-ended answers provided by 2024 respondents on ‘feeling bad’ subsequent of spending time online, a conventional content analysis per the recommendations of Hsieh and Shannon [64] was used. Among the adolescents who in 2024 affirmed feeling bad after spending time on social media (*n*_yes_ = 130; *n*_sometimes_ = 245) or gaming (*n*_yes_ = 63; *n*_sometimes_ = 152), 127 participants (16.5% of all 2024 responders; 34.4% of those who were presented with the social media and/or gaming question) described their experiences in text (mean number of words = 13.4, range 1–135 words). The analysis was initiated by author MN by reading the quotes several times and writing down her initial impressions while highlighting segments that captured key concepts related to the research questions (e.g., mentions of interpersonal interactions). These segments were sorted into thematically coherent clusters, among which contents and coherence was subsequently discussed among the authors. The final (sub-)categories were not mutually exclusive, meaning that participant responses covering many topics have been coded within each.

## Results

### 15-year changes in interpersonal interactions and mental health problems

**Fig. 1:**
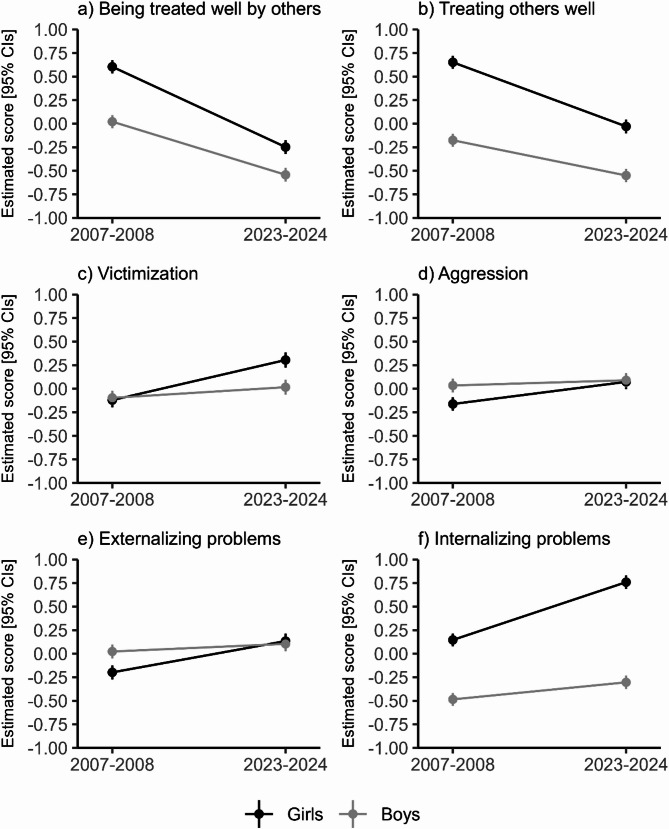
Significant interactions between genders (girls and boys) and period of assessment (2007 − 2008 or 2023 − 2024) in standardized variables related to interpersonal behaviors and externalizing/internalizing problems. Estimates are covaried for whether data was collected at baseline (2007, 2023) or follow-up (2008, 2024)

Table 2 provides a summary of the interaction effects (*p* ≤.018) between period of assessment (i.e., 2007–2008 or 2023–2024) and self-identified gender (*girl* or *boy*) across all outcomes, namely prosocial behaviours (being treated well by others and treating others well), victimization, aggression, and externalizing/internalizing problems. These findings are visualized in Fig. 1. Overall, both girls and boys reported a decrease in prosocial behaviours between the two periods (Δ*β*_girls_ = − 0.43, *p*_bonferroni_ ≤ 0.001; Δ*β*_boys_ = − 0.28, *p*_bonferroni_ ≤ 0.001; Fig. 1a–b), although girls continued to score higher on these measures than did boys. The observed increases in victimization and aggression were significant among girls but not among boys (Δ*β*_girls_ = 0.11–0.21, *p*_bonferroni_ ≤ 0.001; Δ*β*_boys_ = 0.03–0.06, *p*_bonferroni_ ≥ 0.0939; Fig. 1c–d). Externalizing problems only increased significantly among girls (Δ*β*_girls_ = 0.16, *p*_bonferroni_ ≤ 0.001; Δ*β*_boys_ = 0.04, *p*_bonferroni_ = 0.307; Fig. 1e), while internalizing problems increased significantly among both girls and boys (Δ*β*_girls_ = 0.31, *p*_bonferroni_ ≤ 0.001; Δ*β*_boys_ = 0.09, *p*_bonferroni_ ≤ 0.001; Fig. 1f).

**Table 2:** Mixed linear modelling examining differences between 2007 − 2008 and 2023 − 2024 in interpersonal interactions and mental health problems

	Prosocial interactions with others						
	Being treated well by others				Treating others well		
Predictor	*β* (*SE*)	*β*_robust_	*p*		*β* (*SE*)	*β*_robust_	*p*
Intercept	0.58 (0.04)	0.62	< 0.001		0.64 (0.04)	0.66	< 0.001
Assessment (0 = *baseline*, 1 = *follow-up*)	0.04 (0.02)	0.03	0.037		0.03 (0.02)	0.03	0.156
Period (0 = *2007 − 2008*, 1 = *2023 − 2024*)	− 0.85 (0.05)	− 0.87	< 0.001		− 0.68 (0.05)	− 0.68	< 0.001
Gender (0 = *Girls*, 1 = *Boys*)	− 0.58 (0.05)	− 0.59	< 0.001		− 0.83 (0.05)	− 0.85	< 0.001
Period×Gender	0.29 (0.07)	0.28	< 0.001		0.31 (0.07)	0.31	< 0.001
	Negative interactions with others						
	Victimization				Aggression		
	*β* (*SE*)	*β*_robust_	*p*		*β* (*SE*)	*β*_robust_	*p*
Intercept	− 0.11 (0.04)	− 0.21	0.004		− 0.18 (0.04)	− 0.23	< 0.001
Assessment (0 = *baseline*, 1 = *follow-up*)	− 0.01 (0.02)	− 0.01	0.544		0.04 (0.03)	0.02	0.088
Period (0 = *2007 − 2008*, 1 = *2023 − 2024*)	0.43 (0.06)	0.40	< 0.001		0.24 (0.05)	0.16	< 0.001
Gender (0 = *Girls*, 1 = *Boys*)	0.02 (0.05)	0.03	0.657		0.20 (0.05)	0.13	< 0.001
Period×Gender	− 0.31 (0.08)	− 0.32	< 0.001		− 0.18 (0.08)	− 0.14	0.018
	Mental health problems						
	Externalizing problems				Internalizing problems		
	*β* (*SE*)	*β*_robust_	*p*		*β* (*SE*)	*β*_robust_	*p*
Intercept	− 0.20 (0.04)	− 0.27	< 0.001		0.14 (0.04)	0.09	< 0.001
Assessment (0 = *baseline*, 1 = *follow-up*)	0.01 (0.02)	0.01	0.603		0.02 (0.02)	0.02	0.392
Period (0 = *2007 − 2008*, 1 = *2023 − 2024*)	0.33 (0.06)	0.33	< 0.001		0.61 (0.05)	0.68	< 0.001
Gender (0 = *Girls*, 1 = *Boys*)	0.22 (0.05)	0.16	< 0.001		− 0.63 (0.05)	− 0.62	< 0.001
Period×Gender	− 0.25 (0.08)	− 0.20	0.001		− 0.43 (0.07)	− 0.51	< 0.001

Follow-ups were conducted 1 year after baseline (i.e., in 2008 or 2024)

### Bidirectional associations between interpersonal interactions and mental health

As shown in Table 3, the results largely support significant bidirectional associations between interpersonal interactions and mental health problems, with some exceptions. Specifically, victimization and being treated well by others at baseline were significant predictors of externalizing problems (*β*_victimization_ = 0.10–0.18, *β*_treated well_ = − 0.09–-0.17) and internalizing problems (*β*_victimization_ = 0.24–0.31, *β*_treated well_ = -0.20–-0.21). At follow-up, both these interpersonal interactions were predicted by internalizing (|*β| =* 0.20–30) and externalizing problems (*β*_victimization_ = 0.27; *β*_treated well_ = -0.06). Furthermore, treating others well at baseline predicted follow-up internalizing problems (*β =* 0.07–0.10), while at follow-up, treating others well was predicted by baseline externalizing/internalizing problems (*β=* -0.05–-0.07). Finally, aggression showed more specific patterns. Baseline aggression predicted externalizing problems at follow-up (*β =* 0.32–0.34). In turn, aggression at follow-up was predicted by both baseline internalizing problems (*β* = 0.40) and, to a lesser extent, externalizing problems (*β =* 0.05).

**Table 3:** Mixed modelling examining 1-year bidirectional associations between interpersonal interactions to mental health problems.

Fixed effect	Externalizing problems– *R*^2^_marginal_ = 0.21				Internalizing problems– *R*^2^_marginal_ = 0.34		
	β (SE)	β_robust_	*p*		β (SE)	β_robust_	*p*
Intercept	− 0.05 (0.03)	− 0.11	0.112		0.33 (0.03)	0.30	< 0.001
Gender (0 = *Girls*, 1 = *Boys*)	0.07 (0.04)	0.03	0.090		− 0.82 (0.04)	− 0.83	< 0.001
Period (0 = *2007 − 2008*, 1 = *2023 − 2024*)	0.05 (0.04)	0.07	0.234		0.19 (0.04)	0.19	< 0.001
Being treated well by others	− 0.09 (0.03)	− 0.10	0.005		− 0.20 (0.03)	− 0.19	< 0.001
Treating others well	0.02 (0.03)	0.04	0.502		0.07 (0.03)	0.07	0.011
Victimization	0.17 (0.02)	0.16	< 0.001		0.31 (0.02)	0.33	< 0.001
Aggression	0.34 (0.02)	0.34	< 0.001		0.01 (0.02)	0.01	0.528
	Being treated well by others– *R*^2^_marginal_ = 0.22				Treating others well– *R*^2^_marginal_ = 0.20		
	*β* (*SE*)	*β*_robust_	*p*		*β* (*SE*)	*β*_robust_	*p*
Intercept	0.56 (0.03)	0.60	< 0.001		0.58 (0.03)	0.61	< 0.001
Gender (0 = *Girls*, 1 = *Boys*)	− 0.60 (0.04)	− 0.62	< 0.001		− 0.73 (0.04)	− 0.77	< 0.001
Period (0 = *2007 − 2008*, 1 = *2023 − 2024*)	− 0.62 (0.04)	− 0.63	< 0.001		− 0.51 (0.04)	− 0.51	< 0.001
Externalizing problems	− 0.20 (0.02)	− 0.22	< 0.001		− 0.07 (0.02)	− 0.08	0.004
Internalizing problems	− 0.06 (0.02)	− 0.06	0.002		− 0.05 (0.02)	− 0.04	0.016
	Victimization– *R*^2^_marginal_ = 0.21				Aggression– *R*^2^_marginal_ = 0.18		
Intercept	− 0.07 (0.03)	− 0.13	0.020		− 0.07 (0.03)	− 0.13	0.022
Gender (0 = *Girls*, 1 = *Boys*)	0.10 (0.04)	0.10	0.017		0.11 (0.04)	0.09	0.006
Period (0 = *2007 − 2008*, 1 = *2023 − 2024*)	0.10 (0.04)	0.07	0.013		0.03 (0.04)	− 0.02	0.426
Externalizing problems	0.30 (0.02)	0.31	< 0.001		0.05 (0.02)	0.07	0.017
Internalizing problems	0.27 (0.02)	0.26	< 0.001		0.40 (0.02)	0.36	< 0.001

### Predictive value of cybervictimization/cyberaggression for mental health problems in the 2023–2024 cohort

Regarding the relative importance of cybervictimization in 2023 for outcomes in 2024, Table 4 indicates that online interactions contributed with a small, albeit significant, amount to explaining the variance in externalizing and internalizing problems (|Δ|*R*^2^_marginal_ ≤ 0.01). Cybervictimization and cyberaggression at baseline predicted internalizing problems at follow-up (*β*_cybervictimization_ = 0.11; *β*_cyberaggression_ = − 0.09), while baseline internalizing problems predicted both online interactions at follow-up (*β =* 0.28–0.31; *β*_robust_ *=* 0.13–0.18). However, only baseline cybervictimization had a stable, statistically significant association with externalizing problems at follow-up (*β* = 0.13) and vice versa (*β* = 0.16; *β*_robust_ *=* 0.00 for cyberaggression).

**Table 4:** Mixed modelling examining 1-year bidirectional associations between interpersonal interactions including cyberaggression/cybervictimization to mental health problems.

Fixed effect		Externalizing problems				Internalizing problems		
		β (SE)	β_robust_	*p*		β (SE)	β_robust_	*p*
Step 1		*R*^2^_marginal_ = 0.23				*R*^2^_marginal_ = 0.40		
	Intercept	0.01 (0.04)	− 0.03	0.792		0.47 (0.04)	0.46	< 0.001
	Gender (0 = *Girls*, 1 = *Boys*)	0.00 (0.06)	− 0.02	0.986		− 0.92 (0.05)	− 0.95	< 0.001
	Being treated well by others	− 0.18 (0.04)	− 0.19	< 0.001		− 0.21 (0.04)	− 0.21	< 0.001
	Treating others well	0.06 (0.04)	0.07	0.180		0.10 (0.04)	0.10	0.008
	Victimization	0.18 (0.03)	0.17	< 0.001		0.31 (0.03)	0.33	< 0.001
	Aggression	0.35 (0.03)	0.35	< 0.001		0.01 (0.03)	0.01	0.851
Step 2		*R*^2^_marginal_ = 0.24				*R*^2^_marginal_ = 0.40		
ΔStep 1 − Step 2		χ^2^(1) = 14.79, *p* <.001				χ^2^(1) = 1.71, *p* =.005		
	Intercept	0.03 (0.04)	− 0.01	0.487		0.46 (0.04)	0.46	< 0.001
	Gender (0 = *Girls*, 1 = *Boys*)	− 0.03 (0.06)	− 0.05	0.572		− 0.92 (0.05)	− 0.94	< 0.001
	Being treated well by others	− 0.17 (0.04)	− 0.18	< 0.001		− 0.21 (0.04)	− 0.21	< 0.001
	Treating others well	0.05 (0.04)	0.06	0.237		0.10 (0.04)	0.10	0.010
	Victimization	0.10 (0.04)	0.10	0.022		0.24 (0.04)	0.25	< 0.001
	Aggression	0.32 (0.04)	0.31	< 0.001		0.05 (0.04)	0.06	0.180
	Cybervictimization	0.11 (0.04)	0.13	0.007		0.11 (0.04)	0.14	0.004
	Cyberaggression	0.05 (0.04)	0.06	0.173		− 0.09 (0.04)	− 0.11	0.009
		Cybervictimization				Cyberaggression		
		*β* (*SE*)	*β*_robust_	*p*		*β* (*SE*)	*β*_robust_	*p*
		*R*^2^_marginal_ = 0.12				*R*^2^_marginal_ = 0.10		
Intercept		− 0.07 (0.04)	− 0.25	0.088		− 0.10 (0.04)	− 0.31	0.011
Gender (0 = *Girls*, 1 = *Boys*)		0.21 (0.06)	0.19	0.001		0.22 (0.06)	0.13	0.001
Externalizing problems		0.16 (0.03)	0.16	< 0.001		− 0.07 (0.03)	0.00	0.037
Internalizing problems		0.28 (0.03)	0.18	< 0.001		0.31 (0.03)	0.13	< 0.001

### Exploring adolescents’ own narratives of negative online experiences

**Fig. 2:**
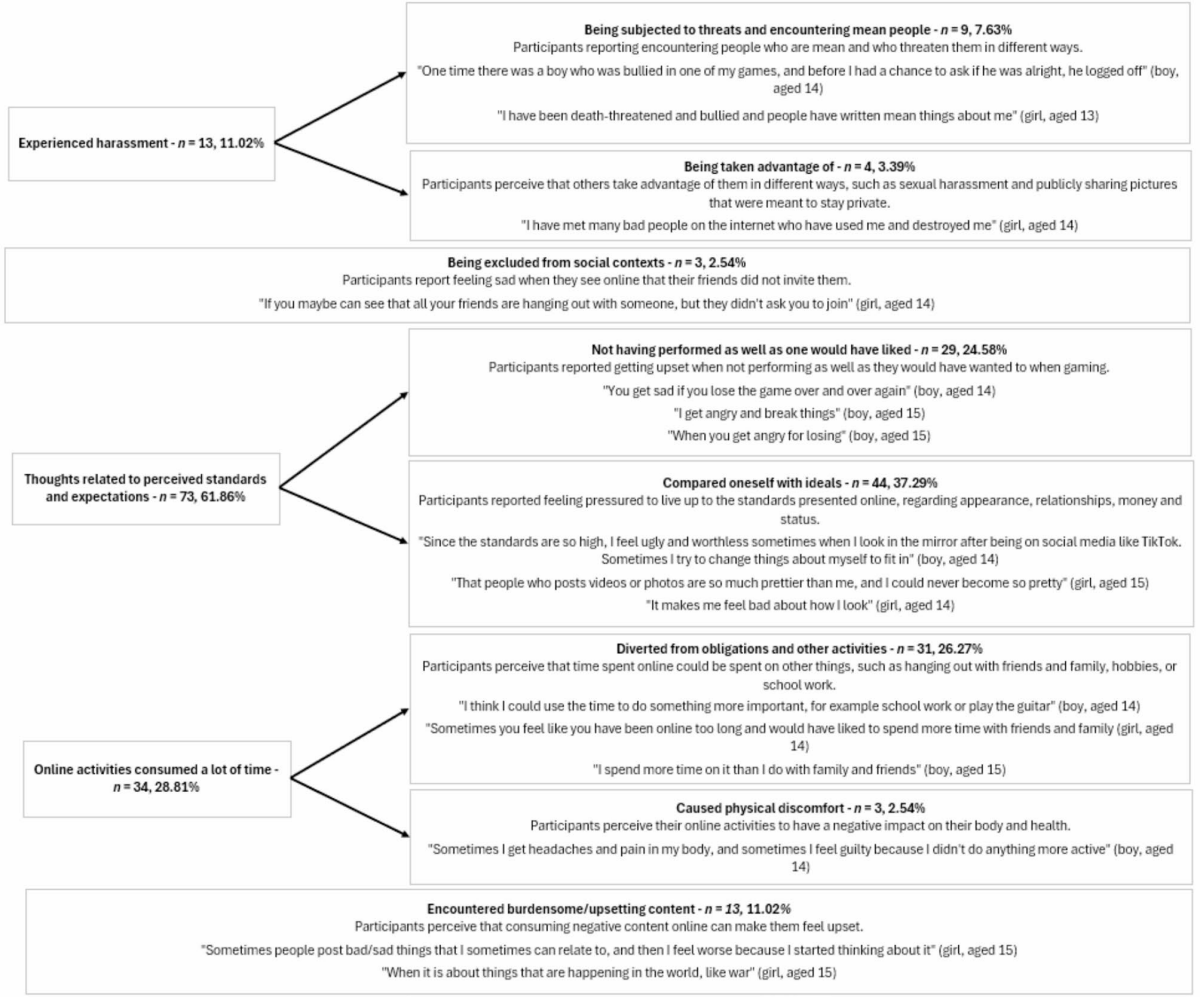
Visualization of the conventional content analysis, showing the categories and subcategories

The categories and subcategories derived from the content analysis are illustrated in Fig. 2. These findings reveal five overarching categories, further divided into six subcategories, which encompass the various reasons adolescents reported ‘feeling bad’ after being online.

‘Feeling bad’ after online interactions was categorized as *experienced harassment* (*n* = 13, 11.02%) and *being excluded from social contexts* (*n* = 3, 2.54%). The remaining categories were not explicitly linked to online interactions and included *thoughts related to perceived standards and expectations* (*n* = 73, 61.86%), *online activities consume a lot of time* (*n* = 34, 28.81%), or *having encountered burdensome/upsetting content* (*n* = 13, 11.02%). Most (92.3%) participants whose answer was categorized as having *experienced harassment* scored above the 50th percentile in cybervictimization, whereas the other categories were more equally spread across percentiles (Supplementary Appendix: Table A8). Fisher’s exact test for count data supported that the number of participants scoring in the 25-50th percentile vs. ≥51st percentile in *experienced harassment* was marginally different from *being excluded from social contexts* (*p* =.071) and significantly different from the other categories (*p* ≤.016).

With regards to experienced harassment in terms of *being subjected to threats and encountering mean people* (*n* = 9, 7.63% of all responders), the statements included having been victimized and threatened, witnessing someone else being victimized, or a perception that people were generally mean and unfiltered online. The other subcategory related to harassment was *being taken advantage of* (*n* = 4, 3.39%). Other aspects of negative online interactions included *social exclusion* (*n* = 3, 2.54%). This related to situations of intentional exclusion within the online space (e.g., exclusion from a group chat or gaming community) and situations with more ambiguous intent, such as encountering pictures of social gatherings where the participant was not invited.

Besides the categories that were more related to interactions with others, managing a perceived failure to live up to standards and expectations (*n* = 73, 61.86%) were present in the data. ‘Feeling bad’ seemed to, in these instances, relate to feelings of disappointment, frustration, worthlessness, and self-doubt. Additionally, guilt was mentioned in relation to obligations and other activities that time could have been spent on instead (*n* = 31, 26.27%), which highlighted how ‘bad feelings’ did not arise from hours spent online in itself, but by evaluating the perceived worthwhileness of alternative activities. Nevertheless, actual time spent was perceived to cause physical discomfort in some adolescents (*n* = 3, 2.54%). Time spent online also increased the likelihood of having encountered burdensome/upsetting content in the context of engaging with others’ lived experiences and news (*n* = 13, 11.02%).

## Discussion

The aim of this study was to better understand how interpersonal behaviours (both in person and online) and adolescent mental health change over time and how they are associated with each other. For this purpose, we first examined 15-year trends in interpersonal behaviours (i.e. aggression, victimization and prosocial behaviours) and externalizing/internalizing problems among Swedish adolescents from 2007 to 2008 to 2023–2024. Second, we explored one-year bidirectional associations between these interpersonal interactions and mental health outcomes, focusing on how both positive (prosocial) and negative (aggressive/victimizing) behaviours mutually relate to mental health over time. Third, we assessed the unique contributions of cybervictimization and cyberaggression to externalizing/internalizing problems in the 2023–2024 cohort. Finally, we contextualized the quantitative findings through a qualitative analysis of open-ended responses from a subsample of adolescents who described online experiences that made them feel bad. By combining longitudinal data with adolescents’ own perspectives, this study offers a more nuanced understanding of the relational and emotional dimensions of adolescent mental health in the digital age.

Our findings indicate that between 2007 and 2024, prosocial behaviours moderately declined among girls and boys, while internalizing problems increased moderately among girls and weakly among boys. Furthermore, victimization, aggression, and externalizing problems increased weakly-moderately only among girls. These findings are consistent with our hypotheses and align with research conducted prior to 2020 [2, 4, 6, 7, 9, 18, 20, 22, 23]. Among these, those who assessed differences between girls and boys found– like this study– that girls tend to score higher in internalizing problems and interpersonal difficulties at baseline as well as follow-up [1, 12, 13], and that increases were particularly pronounced among girls and weak/non-significant for boys [2, 4, 6]. This consistent pattern suggests that adolescents, particularly girls, have faced growing relational challenges and mental health issues over the past 15 years. We also found weak-moderate bidirectional associations between interpersonal interactions and mental health problems in predicting one-year changes. These associations were for victimization and being treated well by others, which had consistent moderate associations to internalizing problems, and weak-moderate associations to externalizing problems. This suggests that both negative and positive interpersonal experiences are reciprocally linked with adolescents’ mental health, implying ongoing cycles where social difficulties and mental health problems may reinforce each other over time– or, conversely, where positive interactions may support improved mental well-being.

The presence of bidirectional effects for these variables, along with similar patterns for other interpersonal behaviours, indicates that neither interpersonal difficulties nor mental health problems can be considered the sole cause of each other. Instead, our findings support the notion of a dynamic, reciprocal relationship between adolescents’ social environments and mental health. These patterns may reflect exo- and macro-level changes not captured in the present study (c.f [14]), such as the rapid rise in social media use (e.g [65]),growing academic and societal pressures (e.g [66]), and heightened awareness and openness around mental health (e.g [67]). Such factors could shape both how adolescents interact with others and how they experience and report psychological distress, underscoring the need for continued, context-sensitive research in this area.

In addition to in-person interactions, we investigated the role of cybervictimization in explaining changes in externalizing and internalizing problems over one year. Consistent with our hypotheses, we found that the additional variance explained by cybervictimization was relatively small compared to that of in-person behaviours. Similarly, when mental health was used to predict changes in online interactions, any significant effects were weaker than for in-person behaviours. Thus, while cybervictimization may be especially detrimental for adolescents already experiencing victimization in person [41, 44–46], it is not sufficient to account for the overall decline in adolescent mental health, nor is deteriorating adolescent mental health sufficient to explain how adolescents act online. The study’s findings support that the primary concern lies in the overall increase in aggression and victimization, including those online. The online context may thus be best understood as an extension of adolescents’ existing areas of interaction rather than a separate relational environment warranting particular concern. Moreover, although we did not assess prosocial behaviours within online contexts, the simultaneous decline in prosocial behaviour scores implies that adolescents may be experiencing less of the protective buffer these positive interactions can provide for mental health.

While cybervictimization did not emerge as a particularly salient factor in explaining internalizing and externalizing problems in the current study, an expanding body of literature indicates that the online context can contribute to mental health challenges in contemporary youth [8, 37, 38, 40, 42]. Although our content analysis was based on only 16.5% of the 2024 respondents, it nevertheless highlights additional phenomena that have become more prevalent in adolescent life since the late 2000s. These categories align with those identified by Maheux et al. [68] suggesting that increased online engagement leads adolescents to more frequently compare themselves with others and societal norms or ideals, encounter distressing content, and struggle with the demands of constant connectivity. In line with these findings, we argue that future research on adolescent online experiences should adopt a more holistic perspective– one that goes beyond interpersonal dynamics such as cybervictimization or cyberaggression. Such a perspective might include adolescents’ engagement with online discourses around norms, ideals, and performance; the extent to which adolescents perceive their time spent online as problematic; and the nature of the content they engage with. Importantly, this approach should also acknowledge that online activity can benefit adolescent mental health by fostering social connectedness, providing entertainment, and facilitating the formation of new friendships [40]. In light of these insights, further refinement of the PANIBI-SF and similar instruments would benefit from incorporating a broader conceptualization of online interactions. For example, the negative dimension of online experiences could be disaggregated into personally experienced, observed, and anticipated cybervictimization. Additionally, future measurement tools should consider contextual factors such as the source of the experience (e.g., friend or stranger), intentionality, severity (e.g., insults or sexual harassment), and public exposure (e.g., private chat or public post), all of which may shape the perceived salience and psychological impact of online interactions.

### Strengths and limitations

A key strength of this study is its longitudinal design, which utilizes data from school cohorts within the same municipality across two time periods (2007–2008 and 2023–2024). This design enables direct comparisons of adolescent interpersonal behaviours and mental health over a 15-year span. Additionally, the integration of quantitative and qualitative data provides a richer, more nuanced perspective.

Nonetheless, several limitations should be noted. First, the response rate in 2023–2024 was lower than that in 2007–2008, which increases the risk of non-random attrition (e.g., students with long-term absenteeism might be underrepresented). Although attrition in school cohort studies tends to have a limited impact on generalizability [69], targeted recruitment strategies and more in-depth data collection methods (e.g., interviews, focus groups) would help capture the experiences of non-responders and reduce potential bias. In addition, participants in 2007–2008 could only identify as either a girl or a boy, preventing the study from addressing experiences among trans and gender-diverse youth– an important gap given their elevated risk for victimization and discrimination [70].

Some limitations regarding the measures used should be noted. This study, like previous research [54, 56], found that the SDQ-s subscales measuring externalizing/internalizing problems showed low internal consistency. As we found evidence of at least metric invariance between periods and other studies support the validity of the SDQ-s in research and clinical settings [56], the low Cronbach’s alpha values might be the result of adequate construct coverage in combination with relatively few items. Nevertheless, future studies about longitudinal change might prefer another operationalization of mental health problems. Furthermore, the PANIBI-SF– our measure of interpersonal behaviours– was developed specifically for this project. Its validity is supported by good psychometric properties (see also [13]), metric invariance across time, and the finding that 92.3% of adolescents who reported negative online experiences scored above the 50th percentile on the cybervictimization subscale (compared with 54% for other categories; see Supplementary Table A8). This suggests stable item interpretation [54] and that any social desirability bias is likely consistent across cohorts. Efforts to reduce such bias included mixing positive (e.g., giving someone a hug) and negative items (e.g., hitting someone) and presenting victimization items prior to those regarding perpetration (see also [21]). Still, as social desirability may particularly affect responses related to aggression [71], further validation is recommended.

Furthermore, only 16.5% of the 2024 respondents answered the open-ended questions, limiting the representativeness and depth of the content analysis. Both the content analysis and the PANIBI-SF focuses solely on negative online experiences, overlooking the potential benefits of online experiences (e.g., interacting with friends, accessing community support and resources, finding information, and engaging in hobbies/entertainment). This is important, as only about a third of adolescents report reduced well-being from social media use [72], while others may experience enhanced mood or connection afterwards [8, 73].

Finally, while the current study provides insights into relationships between interpersonal behaviours and mental health issues, it does not address the reasons that adolescents treat each other differently in 2023–2024 than in 2007–2008. According to ecological systems theory [14], these differences likely reflect changes in broader contexts, such as in the meso- (e.g., school–home dynamics), exo- (e.g., academic pressure, parental stress), and macro-levels (e.g., digitalization, shifting social norms). For instance, increased online communication may reduce face-to-face conflict resolution, and heightened societal focus on achievement may intensify peer competition. To better understand these dynamics, qualitative research that considers structural and contextual influences is needed.

## Conclusion

The results from this study offer several key insights into how interpersonal behaviours– both in-person and online– are linked to adolescent mental health over time.

First, across the 15-year period from 2007 to 2008 to 2023–2024, we observed a moderate decline in prosocial behaviours among girls and boys, plus a weak (boys) and moderate (girls) increase in internalizing problems. Additionally, we observed weak-moderate increases in victimization, aggression, and externalizing problems among girls. These results suggest growing relational challenges and mental health concerns among adolescents, particularly girls.

Second, bidirectional associations were found between interpersonal behaviours and mental health. Victimization consistently predicted higher levels of externalizing/internalizing problems 1 year later, while being treated well by others was linked to lower levels of mental health problems, to a moderate effect in both cases. The reciprocal nature of these associations suggests that neither interpersonal behaviours nor mental health problems alone can be viewed as the root cause; instead, they likely reinforce each other over time, potentially shaped by broader societal or structural changes not addressed in this study.

Third, cybervictimization and cyberaggression made a minor contribution to predicting mental health problems, and vice versa, indicating that in-person behaviours remain the primary drivers of adolescent psychological distress, likely influencing online behaviour as well.

Finally, adolescents’ own narratives revealed that harassment, social exclusion, perceived standards and expectations, time-consuming online activities, and exposure to upsetting content contributed to ‘feeling bad’ after being online. These experiences reflect broader digital phenomena such as heightened social comparison and constant connectivity, which are increasingly shaping adolescents’ emotional lives.

Together, these findings underscore the importance of adopting a holistic perspective in future research– one that captures both the risk and resources present in adolescents’ in-person and online worlds. Understanding how the negative *and* positive interpersonal dynamics associate with mental health and well-being is critical for informing prevention and intervention strategies aimed at supporting today’s youth.

## Electronic supplementary material

Below is the link to the electronic supplementary material.

Supplementary Material 1

## Data Availability

The datasets generated and/or analysed during the current study are not publicly available due to ethical restrictions but are available from the corresponding author on reasonable request.

## Declarations

Consent was obtained from all participants, with passive consent provided by legal guardians for those under 15 years of age. Guardians were informed that they could contact the research leader if they wished to withdraw their child from the study.
