# Supplementary material for: Adolescent Interpersonal Behaviours and Mental Health Across Two Swedish Cohorts: 15-Year Trends and One-Year Bidirectional Associations in a Mixed-Methods Study
Source: Child Adolesc Psychiatry Ment Health. 2025 May 21;19:61. doi: 10.1186/s13034-025-00913-5 (PMC12093687; doi:10.1186/s13034-025-00913-5)
Supplement: Supplementary file 1 — Supplementary Material 1 [file 13034_2025_913_MOESM1_ESM.docx]

## Table A1.

*Items and factor loadings (from confirmatory factor analysis) for PANIBI-SF subscales that focus on how one is treated by others*

| Factor − λ̂* (*SE*) | Period 1: 2007−2008 | |  | Period 2: 2023–2024 | | Swedish (original) | English (translated) |
| --- | --- | --- | --- | --- | --- | --- | --- |
|  | 2007 | 2008 |  | 2023 | 2024 |  |  |
|  |  |  |  |  |  | Hur ofta händer det att… | How often does it happen that … |
| Treated well by others | 0.50 (0.03) | 0.51 (0.03) |  | 0.55 (0.03) | 0.50 (0.03) | … någon ger dig en kram? | … someone gives you a hug? |
|  | 0.80 (0.02) | 0.80 (0.01) |  | 0.80 (0.02) | 0.82 (0.02) | … någon ger dig beröm? | … someone gives you praise? |
|  | 0.70 (0.02) | 0.73 (0.02) |  | 0.82 (0.01) | 0.80 (0.02) | … någon bjuder dig på fest? | … someone invites you to a party? |
| Victimization | 0.86 (0.01) | 0.88 (0.01) |  | 0.90 (0.01) | 0.86 (0.01) | ... någon slår eller sparkar dig? | … someone hits or kicks you? |
|  | 0.72 (0.02) | 0.73 (0.02) |  | 0.69 (0.02) | 0.66 (0.02) | … någon skriker negativa saker till dig? | … someone shouts negative things at you? |
|  | 0.71 (0.03) | 0.60 (0.03) |  | 0.38 (0.04) | 0.37 (0.04) | … någon ger dig fula namn? | … someone calls you bad names? |
|  | 0.67 (0.03) | 0.75 (0.03) |  | 0.78 (0.05) | 0.73 (0.05) | … någon säger elaka saker om dig? | … someone says mean things about you? |
|  | 0.50 (0.03) | 0.52 (0.03) |  | 0.69 (0.05) | 0.80 (0.05) | … någon talar illa bakom ryggen på dig? | … someone talks badly about you behind your back? |
| Cybervictimization |  |  |  | 0.66 (0.03) | 0.61 (0.03) | … någon säger elaka saker till dig eller hotar dig eller kallar dig fula namn på nätet eller när du spelar datorspel online? | … someone say mean things, threaten, or call you bad names on the internet, or when you play computer games online? |
|  |  |  |  | 0.75 (0.02) | 0.74 (0.02) | … någon postar pinsamma videor eller bilder av dig på nätet? | … someone post embarrassing videos or pictures of you on the internet? |
|  |  |  |  | 0.80 (0.02) | 0.82 (0.02) | … någon postar personlig information eller sprider rykten om dig på nätet? | …someone post personal information or spread rumors about you on the internet? |
|  |  |  |  | 0.62 (0.03) | 0.74 (0.02) | … personer du träffar på nätet eller i datorspel utesluter dig. eller behandlar dig som om du inte fanns? | … someone excludes you or treat you like you do not exist on the internet or when playing computer games? |

*Note*. The subscale *cybervictimization* was not included in the data collection for 2007−2008. PANIBI-SF = Positive and Negative Interpersonal Behaviours Inventory−Short Form; *SE* = Standard Error.

## Table A2.

*Items and factor loadings (from confirmatory factor analysis) for PANIBI-SF subscales that focus on how one treats others*

| Factor − λ̂* (*SE*) | Period 1: 2007−2008 | |  | Period 2: 2023–2024 | | Swedish (original) | English (translated) |
| --- | --- | --- | --- | --- | --- | --- | --- |
|  | 2007 | 2008 |  | 2023 | 2024 |  |  |
|  |  |  |  |  |  | Hur ofta händer det att… | How often does it happen that… |
| Treating others well | 0.58 (0.03) | 0.58 (0.03) |  | 0.65 (0.02) | 0.65 (0.02) | … att du ger någon en kram? | … you give someone a hug? |
|  | 0.70 (0.02) | 0.70 (0.02) |  | 0.80 (0.02) | 0.80 (0.02) | … att du ger någon beröm? | … you give someone praise? |
|  | 0.66 (0.02) | 0.66 (0.02) |  | 0.85 (0.01) | 0.85 (0.01) | … att du bjuder någon på fest? | … you invite someone to a party? |
| Aggression | 0.74 (0.02) | 0.74 (0.02) |  | 0.78 (0.02) | 0.78 (0.02) | …att du slår eller sparkar någon? | … you hit or kick someone? |
|  | 0.52 (0.03) | 0.52 (0.03) |  | 0.52 (0.03) | 0.52 (0.03) | … att du skriker negativa saker till någon? | … you shout negative things at someone? |
|  | 0.68 (0.03) | 0.68 (0.03) |  | 0.43 (0.04) | 0.43 (0.04) | … att du ger någon fula namn? | … you call someone bad names? |
|  | 0.69 (0.03) | 0.69 (0.03) |  | 0.72 (0.04) | 0.72 (0.04) | … att du säger elaka saker om någon? | … you say mean things about someone? |
|  | 0.58 (0.03) | 0.58 (0.03) |  | 0.78 (0.04) | 0.78 (0.04) | … att du talar illa bakom ryggen på någon? | … you talk badly about someone behind their back? |
| Cyberaggression |  |  |  | 0.59 (0.03) | 0.59 (0.03) | …att du säger elaka saker till någon eller hotar någon och kallar någon fula namn på nätet eller när du spelar datorspel online? | … you say mean things, threaten, or call someone bad names on the internet, or when you play computer games online? |
|  |  |  |  | 0.75 (0.02) | 0.75 (0.02) | … att du postar pinsamma videor eller bilder av någon på nätet? | … you post embarrassing videos or pictures of someone on the internet? |
|  |  |  |  | 0.72 (0.02) | 0.72 (0.02) | … att du postar personlig information eller sprider rykten om någon på nätet? | … you post personal information or spread rumors about someone on the internet? |
|  |  |  |  | 0.71 (0.02) | 0.71 (0.02) | … att du på nätet eller i datorspel utesluter någon eller behandlar någon som om hen inte fanns? | … you exclude someone, or treat them like they did not exist on the internet or when playing computer games? |

*Note*. The subscale *cyberaggression* was not included in the data collection for 2007−2008. PANIBI-SF = Positive and Negative Interpersonal Behaviours Inventory−Short Form; *SE* = Standard Error.

## Table A3.

*Measurement invariance between 2007−2008 and 2023−2024 of the PANIBI-SF*

|  | Victimization and treated well by others | | | | | | | | |
| --- | --- | --- | --- | --- | --- | --- | --- | --- | --- |
|  | Baseline (2007 vs. 2023) | | | |  | Follow-up (2008 vs. 2024) | | | |
|  | χ^2^ | CFI | RMSEA [95% CI] | SRMR |  | χ^2^ | CFI | RMSEA [95% CI] | SRMR |
| Simple model | 129.11 | .977 | .059 [.049, .069] | .032 |  | 166.59 | .968 | .069 [.059, .078] | .036 |
| 1. Configural | 197.74 | .965 | .071 [.061, .081] | .034 |  | 243.21 | .955 | .081 [.071, .091] | .045 |
| 2. Metric | 278.43 | .949 | .080 [.071, .089] | .053 |  | 295.84 | .945 | .083 [.074, .092] | .051 |
| 3. Scalar | 364.27 | .932 | .087 [.078, .095] | .057 |  | 391.52 | .925 | .091 [.083, .099] | .059 |
| \|Δ\| 1 vs. 2 | 80.69 | .016 | .009 | .019 |  | 52.63 | .010 | .002 | .007 |
| \|Δ\| 2 vs. 3 | 85.85 | .017 | .007 | .003 |  | 95.68 | .020 | .008 | .008 |
|  |  |  |  |  |  |  |  |  |  |
|  | Aggression and treating others well | | | | | | | | |
|  | Baseline (2007 vs. 2023) | | | |  | Follow-up (2008 vs. 2024) | | | |
| Simple model | 191.36 | .952 | .073 [.064, .083] | .036 |  | 166.59 | .968 | .069 [.059, .078] | .036 |
| 1. Configural | 244.60 | .941 | .080 [.071, .090] | .040 |  | 243.21 | .955 | .081 [.071, .091] | .045 |
| 2. Metric | 309.50 | .924 | .085 [.076, .094] | .052 |  | 295.84 | .945 | .083 [.074, .092] | .051 |
| 3. Scalar | 397.34 | .900 | .091 [.083, .099] | .059 |  | 391.52 | .925 | .091 [.083, .099] | .059 |
| \|Δ\| 1 vs. 2 | 64.90 | .017 | .004 | .011 |  | 52.63 | .010 | .002 | .007 |
| \|Δ\| 2 vs. 3 | 87.84 | .024 | .006 | .008 |  | 95.68 | .020 | .008 | .008 |
|  |  |  |  |  |  |  |  |  |  |
|  | Hu & Bentler (1999) | | | |  | Sass (2016) | | | |
| Acceptable fit/metric invariance |  | >.90 | < .06 | < .09 |  |  | Δ<.01 | Δ<.015 | Δ<.03 |
| Good fit/scalar invariance |  | > .95 | < .05 | < .08 |  |  | Δ<.01 | Δ<.015 | Δ<.01 |

*Note*. Cyberaggression and cybervictimization are excluded from testing as these subscales were not included in the 2007−2008 assessment. Models/invariance were not evaluated with the χ^2^-test due to the test’s sensitivity to sample size and non-normality (Hu & Bentler, 1999; Sass, 2016).

CI = Confidence Interval; CFI = Comparative Fit Index; PANIBI-SF = Positive and Negative Interpersonal Behaviours Inventory−Short Form; RMSEA = Root Mean Square Error of Approximation; SRMR = Root Mean Square Residual.

**Table A4.**

*Measurement invariance between 2007−2008 and 2023−2024 of the emotional symptoms and conduct problems subscales of the SDQ-s.*

|  | Baseline (2007 vs. 2023) | | | |  | Follow-up (2008 vs. 2024) | | | |
| --- | --- | --- | --- | --- | --- | --- | --- | --- | --- |
| Model | χ^2^ | CFI | RMSEA [95% CI] | SRMR |  | χ^2^ | CFI | RMSEA [95% CI] | SRMR |
| 1. Configural | 393.49 | .880 | .075 [.068, .082] | .062 |  | 399.32 | .875 | .076 [.069, .084] | .061 |
| 2. Metric | 413.51 | .876 | .072 [.065, .079] | .065 |  | 423.55 | .869 | .074 [.067, .081] | .065 |
| 3. Scalar | 465.89 | .860 | .073 [.067, .079] | .066 |  | 519.17 | .836 | .079 [.072, .085] | .069 |
| \|Δ\| 1 vs. 2 | 20.02 | .004 | .003 | .003 |  | 24.24 | .006 | .002 | .004 |
| \|Δ\| 2 vs. 3 | 52.38 | .016 | .001 | .001 |  | 95.62 | .033 | .005 | .004 |

*Note*. Measurement invariance was not evaluated with the χ^2^-test due to the test’s sensitivity to sample size and non-normality (Sass, 2016).

CI = Confidence Interval; CFI = Comparative Fit Index; RMSEA = Root Mean Square Error of Approximation; SDQ-s = Strength and Difficulties Questionnaire – self-report version; SRMR = Root Mean Square Residual.

## Table A5.

*Mixed linear modelling examining differences between 2007−2008 and 2023−2024 in interpersonal behaviours and externalizing/internalizing difficulties, while controlling for foreign background.*

|  | Prosocial interactions with others | | | | | | |
| --- | --- | --- | --- | --- | --- | --- | --- |
|  | Being treated well by others | | |  | Treating others well | | |
| Predictor | *β* (*SE*) | *β*_robust_ | *p* |  | *β* (*SE*) | *β*_robust_ | *p* |
| Intercept | .64 (.04) | .67 | <.001 |  | .67 (.04) | .70 | <.001 |
| Foreign background (0 = *no*, 1 = *yes*) | -.01 (.04) | .01 | .866 |  | .06 (.04) | .05 | .184 |
| Assessment (0 = *baseline*, 1 = *follow-up*) | -.06 (.04) | -.06 | .131 |  | -.04 (.04) | -.06 | .237 |
| Period (0 = *2007−2008*, 1 = *2023−2024*) | -.85 (.05) | -.87 | <.001 |  | -.70 (.05) | -.70 | <.001 |
| Gender (0 = *Girls*, 1 = *Boys*) | -.59 (.05) | -.59 | <.001 |  | -.83 (.05) | -.86 | <.001 |
| Period×Gender | .29 (.07) | .29 | <.001 |  | .31 (.07) | .32 | <.001 |
|  | Negative interactions with others | | | | | | |
|  | Victimization | | |  | Aggression | | |
|  | *β* (*SE*) | *β*_robust_ | *p* |  | *β* (*SE*) | *β*_robust_ | *p* |
| Intercept | -.12 (.04) | -.22 | .005 |  | -.16 (.04) | -.22 | <.001 |
| Foreign background (0 = *no*, 1 = *yes*) | -.05 (.05) | -.06 | .308 |  | -.02 (.05) | -.04 | .701 |
| Assessment (0 = *baseline*, 1 = *follow-up*) | .02 (.04) | .02 | .645 |  | .01 (.04) | .01 | .858 |
| Period (0 = *2007−2008*, 1 = *2023−2024*) | .43 (.06) | .41 | <.001 |  | .24 (.06) | .17 | <.001 |
| Gender (0 = *Girls*, 1 = *Boys*) | .03 (.05) | .04 | .623 |  | .19 (.05) | .13 | <.001 |
| Period×Gender | -.31 (.08) | -.32 | <.001 |  | -.18 (.08) | -.15 | .018 |
|  | Mental health problems | | | | | | |
|  | Externalizing problems | | |  | Internalizing problems | | |
|  | *β* (*SE*) | *β*_robust_ | *p* |  | *β* (*SE*) | *β*_robust_ | *p* |
| Intercept | -.22 (.04) | -.29 | <.001 |  | .17 (.04) | .12 | <.001 |
| Foreign background (0 = *no*, 1 = *yes*) | .02 (.05) | .02 | .611 |  | -.18 (.04) | -.19 | <.001 |
| Assessment (0 = *baseline*, 1 = *follow-up*) | .03 (.04) | .04 | .468 |  | .00 (.04) | .00 | .965 |
| Period (0 = *2007−2008*, 1 = *2023−2024*) | .32 (.06) | .32 | <.001 |  | .65 (.05) | .71 | <.001 |
| Gender (0 = *Girls*, 1 = *Boys*) | .22 (.05) | .17 | <.001 |  | -.63 (.05) | -.62 | <.001 |
| Period×Gender | -.26 (.08) | -.22 | .001 |  | -.43 (.07) | -.51 | <.001 |

*Note*. Follow-ups were conducted 1 year after baseline (i.e., in 2008 or 2024).

## Table A6.

*Mixed modelling examining associations between interpersonal interactions to mental health problems at follow-up 1 year later, while controlling for foreign background.*

| Predictor | | Externalizing problems –  *R*^2^_marginal_ = .21 | | |  | Internalizing problems –  *R*^2^_marginal_ = .35 | | |
| --- | --- | --- | --- | --- | --- | --- | --- | --- |
|  |  | *β* (*SE*) | *β*_robust_ | *p* |  | *β* (*SE*) | *β*_robust_ | *p* |
| Intercept | | -.06 (.03) | -.11 | .088 |  | .35 (.03) | .33 | <.001 |
| Foreign background (0 = *no*, 1 = *yes*) | | .02 (.04) | .01 | .633 |  | -.16 (.04) | -.16 | <.001 |
| Gender (0 = *Girls*, 1 = *Boys*) | | .06 (.04) | .03 | .110 |  | -.83 (.04) | -.83 | <.001 |
| Period (0 = *2007−2008*,  1 = *2023−2024*) | | .04 (.04) | .06 | .370 |  | .22 (.04) | .22 | <.001 |
| Being treated well by others | | -.08 (.03) | -.10 | .008 |  | -.19 (.03) | -.19 | <.001 |
| Treating others well | | .02 (.03) | .03 | .595 |  | .07 (.03) | .07 | .020 |
| Victimization | | .17 (.02) | .16 | <.001 |  | .31 (.02) | .33 | <.001 |
| Aggression | | .34 (.02) | .34 | <.001 |  | .01 (.02) | .01 | .496 |
|  |  | 2023–2024 sample only | | | | | | |
|  |  | Externalizing problems | | |  | Internalizing problems | | |
|  |  | *β* (*SE*) | *β*_robust_ | *p* |  | *β* (*SE*) | *β*_robust_ | *p* |
| **Step 1** | | *R*^2^_marginal_ = .23 | | |  | *R*^2^_marginal_ = .41 | | |
|  | Intercept | -.02 (.05) | -.05 | .657 |  | .55 (.04) | .55 | <.001 |
|  | Foreign background (0 = *no*, 1 = *yes*) | .06 (.06) | .05 | .288 |  | -.21 (.05) | -.22 | <.001 |
|  | Gender (0 = *Girls*, 1 = *Boys*) | -.01 (.06) | -.02 | .931 |  | -.93 (.05) | -.95 | <.001 |
|  | Being treated well by others | -.17 (.04) | -.18 | <.001 |  | -.22 (.04) | -.21 | <.001 |
|  | Treating others well | .05 (.04) | .06 | .262 |  | .09 (.04) | .10 | .013 |
|  | Victimization | .18 (.03) | .18 | <.001 |  | .31 (.03) | .33 | <.001 |
|  | Aggression | .35 (.03) | .35 | <.001 |  | .01 (.03) | .01 | .735 |
| **Step 2** | | *R*^2^_marginal_ = .24 | | |  | *R*^2^_marginal_ = .41 | | |
| **ΔStep 1−Step 2** | | χ^2^(1) = 13.91, *p* < .001 | | |  | χ^2^(1) = 1.01, *p* = .007 | | |
|  | Intercept | .00 (.05) | -.03 | .995 |  | .55 (.04) | .54 | <.001 |
|  | Foreign background (0 = *no*, 1 = *yes*) | .05 (.06) | .04 | .364 |  | -.21 (.05) | -.21 | <.001 |
|  | Gender (0 = *Girls*, 1 = *Boys*) | -.04 (.06) | -.05 | .541 |  | -.93 (.05) | -.95 | <.001 |
|  | Being treated well by others | -.16 (.04) | -.16 | <.001 |  | -.22 (.04) | -.21 | <.001 |
|  | Treating others well | .04 (.04) | .05 | .334 |  | .09 (.04) | .09 | .018 |
|  | Victimization | .10 (.04) | .10 | .016 |  | .24 (.04) | .24 | <.001 |
|  | Aggression | .31 (.04) | .31 | <.001 |  | .05 (.04) | .06 | .181 |
|  | Cybervictimization | .11 (.04) | .12 | .010 |  | .11 (.04) | .14 | .004 |
|  | Cyberaggression | .05 (.04) | .06 | .181 |  | -.08 (.04) | -.10 | .020 |

## Table A7.

*Mixed modelling examining associations between mental health problems to interpersonal interactions at follow-up 1 year later, while controlling for foreign background.*

|  | Prosocial interactions with others | | | | | | |
| --- | --- | --- | --- | --- | --- | --- | --- |
|  | Being treated well by others –  *R*^2^_marginal_ = .22 | | |  | Treating others well –  *R*^2^_marginal_ = .21 | | |
| Predictor | *β* (*SE*) | *β*_robust_ | *p* |  | *β* (*SE*) | *β*_robust_ | *p* |
| Intercept | .57 (.03) | .61 | <.001 |  | .58 (.03) | .61 | <.001 |
| Foreign background (0 = *no*, 1 = *yes*) | -.04 (.05) | -.02 | .359 |  | .06 (.05) | .05 | .211 |
| Gender (0 = *Girls*, 1 = *Boys*) | -.61 (.04) | -.63 | <.001 |  | -.75 (.04) | -.78 | <.001 |
| Period (0 = *2007−2008*, 1 = *2023−2024*) | -.61 (.04) | -.62 | <.001 |  | -.52 (.04) | -.52 | <.001 |
| Externalizing problems | -.21 (.02) | -.22 | <.001 |  | -.07 (.02) | -.09 | .001 |
| Internalizing problems | -.06 (.02) | -.05 | .005 |  | -.04 (.02) | -.04 | .037 |
|  | Negative interactions with others | | | | | | |
|  | Victimization –  *R*^2^_marginal_ = .21 | | |  | Aggression –  *R*^2^_marginal_ = .18 | | |
|  | *β* (*SE*) | *β*_robust_ | *p* |  | *β* (*SE*) | *β*_robust_ | *p* |
| Intercept | -.07 (.03) | -.13 | .025 |  | -.07 (.03) | -.12 | .023 |
| Foreign background (0 = *no*, 1 = *yes*) | -.01 (.04) | -.03 | .845 |  | .02 (.04) | -.01 | .624 |
| Gender (0 = *Girls*, 1 = *Boys*) | .10 (.04) | .10 | .010 |  | .10 (.04) | .08 | .008 |
| Period (0 = *2007−2008*, 1 = *2023−2024*) | .09 (.04) | .08 | .021 |  | .03 (.04) | -.01 | .480 |
| Externalizing problems | .30 (.02) | .31 | <.001 |  | .05 (.02) | .07 | .021 |
| Internalizing problems | .27 (.02) | .27 | <.001 |  | .40 (.02) | .37 | <.001 |
|  | Negative interactions with others online (2023–2024 sample only) | | | | | | |
|  | Cybervictimization –  *R*^2^_marginal_ = .12 | | |  | Cyberaggression –  *R*^2^_marginal_ = .10 | | |
|  | *β* (*SE*) | *β*_robust_ | *p* |  | *β* (*SE*) | *β*_robust_ | *p* |
| Intercept | -.11 (.05) | -.26 | .021 |  | -.15 (.05) | -.32 | .002 |
| Foreign background (0 = *no*, 1 = *yes*) | .09 (.06) | .03 | .103 |  | .11 (.06) | .02 | .055 |
| Gender (0 = *Girls*, 1 = *Boys*) | .22 (.06) | .19 | .001 |  | .22 (.06) | .13 | <.001 |
| Externalizing problems | .16 (.04) | .16 | <.001 |  | -.06 (.03) | .00 | .072 |
| Internalizing problems | .27 (.03) | .17 | <.001 |  | .30 (.03) | .13 | <.001 |

## Table A8.

*Distributions of categories derived from content analysis across different percentiles of cybervictimization*

|  |  | Cybervictimization | | | | | | | | | | |
| --- | --- | --- | --- | --- | --- | --- | --- | --- | --- | --- | --- | --- |
| Category | *N* | 25−50^th^ percentile | | |  | 51−75^th^ percentile | | |  | 76−100^th^ percentile | | |
|  |  | *n* | % within category | % within percentile |  | *n* | % within category | % within percentile |  | *n* | % within category | % within percentile |
| Having experienced harassment | 13 | 1 | 7.69 | 1.75 |  | 6 | 46.15 | 17.65 |  | 6 | 46.15 | 15.00 |
| Been excluded from social contexts | 3 | 2 | 66.66 | 3.51 |  | 0 | - | - |  | 1 | 33.33 | 2.50 |
| Thoughts related to perceived standards and expectations | 73^a^ | 31 | 42.47 | 54.39 |  | 16 | 21.92 | 47.06 |  | 23 | 31.51 | 57.50 |
| Online activities consumed a lot of time | 34^a^ | 16 | 47.06 | 28.07 |  | 9 | 26.47 | 26.47 |  | 8 | 23.53 | 20.00 |
| Encountered burdensome/upsetting content | 13^a^ | 7 | 53.85 | 12.28 |  | 3 | 23.08 | 8.82 |  | 2 | 15.38 | 5.00 |

*Note*. All participants who provided text answers scored above the 25^th^ percentile in cybervictimization as measured by the Positive and Negative Interpersonal Behaviours Inventory – Short Form.

^a^ Some participants did not respond to the cybervictimization items, and therefore, have not been assigned a percentile.
